# A survey of Canadian men’s mental health in the workplace

**DOI:** 10.1080/28324765.2024.2313870

**Published:** 2024-03-01

**Authors:** Paul Sharp, John L. Oliffe, David Kealy, Simon M. Rice, Zac E. Seidler, John S. Ogrodniczuk

**Affiliations:** a School of Nursing, University of British Columbia, Vancouver, Canada; b Department of Psychiatry, University of British Columbia, Vancouver, Canada; c Department of Nursing, The University of Melbourne, Melbourne, Australia; d Orygen, Melbourne, Australia; e Centre for Youth Mental Health, The University of Melbourne, Melbourne, Australia; f Movember, Melbourne, Australia

**Keywords:** mental health, men’s health, gender, workplace, masculinity

## Abstract

Work plays a central role in many men’s lives and can be a major contributor to mental health. The current study aim was to examine the prevalence and nature of Canadian men’s mental health challenges at work, including diverse indicators of mental health and workplace characteristics. Participants (*N* = 451) were men (*M* = 49.97 years; *SD* = 14.99) employed in British Columbia, Canada, who were recruited via a market research online panel. Questionnaires were used to collect men’s mental health data (e.g. depression, loneliness), work-related health (e.g. burnout, bullying), and workplace performance (e.g. presentism, absenteeism). Findings revealed high rates of hazardous drinking (36%), depression (22%), suicidal/self-injury ideation (18%), and anxiety (14%). Many participants reported that they frequently experienced occupational burnout, and 18% reported that personal problems significantly impaired their work. Of concern, approximately 53% indicated that they keep feelings to themselves and 45% reported that they would prefer not to talk about their problems. Important factors related to mental health symptoms were identified (e.g. age, distress concealment, male-dominated workplace). These findings highlight the need for policy makers and employers to take immediate action to address men’s mental health challenges by developing initiatives that promote men’s mental health in the workplace.

## Introduction

Across the life course, men spend an average of 42 years in the labour force (Carrière & Mérette, 2021). Paid work has a predominant role in many men’s lives and is deeply embedded in masculine identities and self-worth with strong connections to their mental health (Oliffe & Han, 2014). Many men define themselves and measure their worth on work performativities and their fit with traditional masculine family provider and protector roles (Kilmartin, 2007). Norms prescribing men’s hard work ethic, corporate commitment, competitiveness, and self-sacrifice are celebrated and replicated in many masculine work cultures (Berdahl et al., 2018; Boettcher et al., 2019). Yet men who align with traditional provider roles and work-role orientations often experience poorer mental health (Kilian et al., 2020). For example, Lashewicz et al. (2020) reported that expectations of men’s productivity carried mental health risks which precipitated anxiety and depression when they were not optimally working to their capacity. Social pressures to prioritise work over their personal lives can similarly expose men to risk for poor mental health, social isolation and inhibit men’s self-disclosures and help-seeking (Mahalik & Dagirmanjian, 2019; Robertson et al., 2016).

The relationship between work and men’s mental health is, however, complex and bi-directional. While employment is generally considered a protective factor for mental health (Hergenrather et al., 2015), the context, setting and nature of men’s work influences their mental health. Workplace predictors for depression and anxiety include low social support from peers and/or managers, high workload, job strain, effort—reward imbalance, and job insecurity (Stansfeld & Candy, 2006; Woo & Postolache, 2008). Male-dominated workplaces have been associated with a higher prevalence of mental health problems (Roche et al., 2016), which is attributable, in part, to traditionally normed masculine attitudes and values being upheld and policed within these workplaces (Milner et al., 2018; Oliffe et al., 2017). Competitive masculine climates can also normalise negative workplace environments that include workplace harassment, threats, and bullying (Berdahl et al., 2018), further increasing risk for mental ill-health. Poor mental health is a leading contributor of reduced productivity at work (Allen et al., 2018), as well as burnout and early retirement (Demerouti et al., 2009; Doshi et al., 2008). Furthermore, poor mental health related to work-related stresses and compromised work–life balance can contribute to considerable strain on men’s intimate partner relationships (Barnett et al., 1992; Cassino & Besen-Cassino, 2022). Considered together, it is clear that the relationship between men’s mental health and the workplace is multi-factorial, compounding the negative impacts on men’s mental health and performance (Demerouti et al., 2009).

Previous studies of men’s workplace mental health have focused on specific male-dominated industries (e.g., construction), and have not considered men’s mental health in the workplace from a broader perspective (Roche et al., 2016; Seaton et al., 2017). As such, the current study aim was to examine the prevalence and nature of Canadian men’s mental health challenges at work, including diverse indicators of mental health and workplace characteristics. A better understanding of men’s workplace mental health can inform the development of public health policies and tailored workplace mental health interventions.

## Methods

### Sample and recruitment procedures

Following University ethics approval (H21–01536), respondents were sourced from an online panel market research provider and screened to ensure they met eligibility requirements (i.e., 19 years and older, online access, able to read English, employed full-time or part-time in British Columbia, Canada). Panellists with profiles that met the eligibility criteria were invited to participate in the study via email and notification on the company’s app (typical response rate of 10–15%). The survey topic was not disclosed in the initial invitation, and only potential respondents who went to the survey introduction page were advised that men’s mental health in the workplace was the study focus. Four hundred and fifty-one men who met the eligibility criteria and provided informed consent were included in the sample. Respondents received an honorarium for their participation. The online survey was administered during May–June 2021.

### Survey questionnaire

A comprehensive online questionnaire was developed which included standardised, validated measures as well as questions about participants’ demographics and employment. Self-perceived health was measured using a single item (“In general, would you say your health is … ”) rated from 1 (excellent) to 5 (poor).

Depression was assessed using the 9-item Patient Health Questionnaire (PHQ-9; Kroenke et al., 2001). Total scores (range 0–27) were calculated with higher scores representing greater depressive symptoms and respondents were classified according to their potential depression severity as none (0–4), mild (5–9), moderate (10–14), moderately severe (15–19), and severe (20–27) (Kroenke et al., 2001). Internal consistency of the scale in the current study was high (*α* = .92).

Suicidal/self-injury ideation was assessed using item 9 of the PHQ-9 (“Thoughts that you would be better off dead or of hurting yourself in some way”), with possible values ranging from 0, “Not at all”, to 3, “nearly every day” (Simon et al., 2013).

Hopelessness was measured using the 2-item Brief-H-Neg (Fraser et al., 2014). Respondents indicate agreement on a five-point scale (range 2–10) and higher scores indicate higher hopelessness. Internal consistency of the scale in the current study was satisfactory (*α* = .75).

Anxiety was assessed using the 7-item General Anxiety Disorder scale (GAD-7; Spitzer et al., 2006). Total scores (range 0–21) were calculated, with scores of 10 or greater indicating moderate anxiety suggestive of generalised anxiety disorder, and categorised as none to minimal (0–4), mild (5–9), moderate (10–14), or severe (15–21). Internal consistency of the scale in the current study was high (*α* = .92).

Hazardous alcohol use was assessed using the 3-item Alcohol Use Disorders Identification Test—Consumption scale (AUDIT-C; Bush et al., 1998), with a score of 4 or more indicating men’s hazardous drinking. Internal consistency of the scale in the current study was moderate (*α* = .65).

Loneliness was assessed using the 3-item UCLA Loneliness Scale (UCLA-3; Hughes et al., 2004), with scores ranging from 3 to 9, and higher scores (6 or greater) indicating loneliness. Internal consistency of the scale in the current study was moderate (*α* = .68).

Somatic symptom burden was measured using the 8-item Somatic Symptom Scale (SSS-8; Gierk et al., 2014). Total scores (range 0–32) were calculated and categorised as no to minimal (0–3), low (4–7), medium (8–11), high (12–15), and very high (16–32). Internal consistency of the scale in the current study was high (*α* = .82).

Anger was assessed using the PROMIS Emotional Distress scale (PROMIS-5; Pilkonis et al., 2011), with raw scores ranging from 5 to 25, and high scores indicating higher levels of anger. Raw scores were used to categorised participants’ level of anger as none to slight (5–13), mild (14–15), moderate (16–20), or severe (21–25). Internal consistency of the scale in the current study was moderate (α = .65).

The four-item short form WHO Health and Work Performance Questionnaire (HPQ; Kessler et al., 2003) was used to assess working hours (actual and expected), absenteeism, and presenteeism. Four items from the Copenhagen Psychosocial Questionnaire-III (COPSOQ-III; Burr et al., 2019) were used to assess the frequency of bullying, physical violence, threats of violence, and sexual harassment at work in the last 12 months on a 5-point scale from “yes, daily” to “no”. Participants rated the extent to which they considered their workplace to be male dominated on a 5-point scale from “not at all” to “completely”.

Two items from the Workplace Outcome Suite (Lennox et al., 2018) were used to assess personal problems impacting work (“My personal problems kept me from concentrating on my work”) and dread (“I dread going to work”) on a 5-point scale from “strongly disagree” to “strongly agree”. Burnout frequency was measured using a single item (“I feel burned out from my work”) from the Maslach Burnout Inventory (Maslach et al., 1997) on a 7-point scale ranging from “never” to “every day”.

A 3-item brief version of the Distress Disclosure Index (DDI; Cox et al., 2020; Kahn et al., 2012) was used to assess distress concealment (“I prefer not to talk about my problems”, When I feel depressed or down, I tend to keep those feelings to myself”, “When I’m distressed, I do not tell anyone”). Items were summed (range 3–15) with lower scores indicating greater concealment of distress. Internal consistency of the scale in the current study was high (α = .86).

### Statistical analysis

Analyses were undertaken using SPSS version 27. Quality of the data collected was ensured by checking for duplication in IP addresses, evaluating completion times, and screening for response biases (e.g., non-differentiated responding). Descriptive statistics were used to characterise the sample including means and standard deviations (*SD*) for continuous variables and frequencies and percentages for categorical variables. Spearman’s correlations were used to examine bivariate associations between select personal characteristics (i.e., age, income, employment status, the extent to which a workplace is perceived to be male-dominated, and distress concealment) and mental health indicators (e.g., depression, anxiety, burnout frequency, suicidal/self-injury ideation). Independent sample t-tests were used to examine the relationship between workplace violence, threats, and/or harassment and key mental health indicators.

## Results

### Sample description

The 451 respondents ranged in age from 18 to 87 years (*M* = 49.97; SD = 14.99) and were predominantly white (66.3%; *n* = 299), educated beyond high school (89.6%; *n* = 404), employed full-time (69.8%; *n* = 315), with a personal income ≥$50,000 CAD (65.2%; *n* = 294). Participant characteristics are presented in Table 1. Industry of work varied across 19 response options, with the highest proportion of men working in professional, scientific, and technical services (15.5%, *n* = 70), followed by retail and wholesale (12.9%; *n* = 58). Geographic region varied across the Fraser (34.1%; *n* = 154), Vancouver Costal (35.7%; *n* = 161), Vancouver Island (14.4%; *n* = 65), Interior (11.1%; *n* = 50), and Northern (4.7%; *n* = 21) Health Authorities, roughly proportional to the population size, with a slight overrepresentation of Vancouver Coastal residents (BC Stats, 2022).

**Table 1.** Sociodemographic characteristics of study participants (*N* = 451)

Characteristic	*n* (%)
**Age**	
18–24 years	20 (4.4)
25–35 years	73 (16.2)
36–50 years	117 (25.9)
51–64 years	160 (35.5)
65+ years	81 (18.0)
**Sexuality**	
Heterosexual	392 (86.9)
Gay/bisexual/other	59 (13.1)
**Education**	
Some or all High School	47 (10.4)
Diploma or Certificate	101 (22.4)
Some College	69 (15.3)
Bachelor’s Degree	149 (33.0)
Post Graduate Degree	85 (18.8)
**Relationship status**	
Single	108 (23.9)
Married/In a relationship	343 (76.1)
**Employment status**	
Full time	315 (69.8)
Part time	136 (30.2)
**Income ($CAD)**	
<50,000	157 (34.8)
≥50,000	294 (65.2)
**Male-dominated work environment**	
Not at all	163 (36.1)
Somewhat	116 (25.7)
Moderately	78 (17.3)
A lot	59 (13.1)
Completely	35 (7.8)

### Mental health indicators

While most respondents reported their general health to be good to excellent (82.7%, *n* = 373), the mean PHQ-9 score was 5.65 (*SD* = 5.94), corresponding to mild depressive symptoms. Over one fifth (22.4%; *n* = 101) of the sample scored above the threshold for probable major depression (score ≥10) and 18.2% (*n* = 82) experienced thoughts of suicide or self-injury in the last two weeks. Relatedly, more than 20% of men expressed hopelessness about the future, feeling that it was impossible that things could change for the better (*n* = 91) or that they could achieve their goals (*n* = 99). The mean Brief-H-Neg score was 4.75 (*SD* = 2.15).

The mean GAD-7 score was 4.31 (*SD* = 4.86) and 13.7% (*n* = 62) of men’s scores indicated moderate anxiety suggestive of generalised anxiety disorder (score ≥10). Over one fifth of respondents (20.8%; *n* = 94) experienced high to very high somatic symptom burden, and 15.6% (*n* = 70) scored above the threshold for moderate-severe anger. Over one third (36.4%; *n* = 164) of participants reported behaviours indicating hazardous drinking, and 30.4% (*n* = 137) reported being lonely.

Overall, almost two thirds (62.1%; *n* = 280) of respondents met criteria for one or more mental health-related challenges (i.e., depression, anxiety, suicidal/-self-injury ideation, loneliness, or hazardous drinking). Respondents mental health outcomes are presented in Table 2.

**Table 2.** Men’s mental health indicators

Variable	Mean (*SD*)	Response Item, *n* (%)				
General health	–	Poor	Fair	Good	Very Good	Excellent
		15 (3.3)	63 (14.0)	180 (39.9)	145 (32.2)	48 (10.6)
Depression	5.65 (5.94)	None to minimal	Mild	Moderate	Moderately severe	Severe
		247 (54.8)	103 (22.8)	61 (13.5)	20 (4.4)	20 (4.4)
Somatic symptoms	7.03 (5.61)	None to minimal	Low	Medium	High	Very high
		144 (31.9)	135 (29.9)	78 (17.3)	52 (11.5)	42 (9.3)
Suicidal/self-injury ideation	–	Not at all	Several days	More than half the days	Nearly every day	
		369 (81.8)	51 (11.3)	17 (3.8)	14 (3.1)	
Anxiety	4.31 (4.86)	None to minimal	Mild	Moderate	Severe	
		292 (64.7)	97 (21.5)	37 (8.2)	25 (5.5)	
Anger	10.82 (4.24)	None to slight	Mild	Moderate	Severe	
		291 (64.5)	90 (20.0)	63 (14.0)	7 (1.6)	
Loneliness	4.69 (1.83)	No	Yes			
		314 (69.6)	137 (30.4)			
Hazardous drinking	2.88 (2.34)	No	Yes			
		287 (63.6)	164 (36.4)			

Participants reported high levels of distress concealment (*M* = 8.11, *SD* = 2.97) indicating that they keep feelings to themselves (53.0%; *n* = 239), that they would prefer not to talk about their problems (44.6%, *n* = 201), and that they do not tell anyone when they are distressed (39.9%, *n* = 180).

### Work-related factors

Respondents reported working more hours in the last 7 days (*M* = 37.79; *SD* = 18.08) than their employer expected them to work in a typical week (*M* = 36.21; *SD* = 15.20), *t*(432) = 2.57, *p* = .01, with men working an average 5.80 (*SD* = 46.92) hours extra per month, indicating minimal evidence of absenteeism across the sample. However, men’s average rating of their overall job performance in the past month was 76.0%, suggesting that a proportion of men were going to work despite not functioning at their optimal or typical level of productivity (i.e., presenteeism). Indeed, 17.7% (*n* = 80) of participants reported that personal problems significantly impaired their work, and 17.7% (*n* = 80) reported that they dread going to work. Only 15.3% (*n* = 69) of participants reported that they had never experienced burnout from work, whereas 45.0% experienced burnout annually (*n* = 203), 30.2% monthly (*n* = 136), 15.9% weekly (*n* = 72), and 5.5% daily (*n* = 25). Approximately 1 in 5 respondents (21.5%; *n* = 97) experienced at least one form of violence or harassment in the workplace, including bullying (16.0%; *n* = 72), physical violence (6.4%; *n* = 29), threats of violence (8.6%; *n* = 39), and/or sexual harassment (7.8%; *n* = 35).

### Factors associated with men’s mental health in the workplace

As shown in Table 3, several significant bivariate associations emerged from the correlation analyses between men’s personal characteristics and mental health indicators. Age was found to have negative associations with depression (*r* = -.18, *p* < .001), suicidal/self-injury ideation (*r* = -.20, *p* < .001), anxiety (*r* = -.20, *p* < .001), loneliness (*r* = -.21, *p* < .001), and anger (*r* = -.14, *p* < .01), indicating greater symptoms among younger men. Income was negatively correlated with hopelessness (*r* = -.14, *p* = .003) and hazardous drinking (*r* = .16, *p* < .001), with greater hopelessness experienced at lesser incomes, and more hazardous drinking occurring with increasing income. The extent to which a workplace was male-dominated was associated with depression (*r* =.12, *p*=.01) and somatic symptoms (*r* = .13, *p* = .006), with greater symptoms among men in more male-dominated workplaces. Distress concealment was correlated with depression (*r* = .36, *p* < .001), suicidal/self-injury ideation (*r* = .23, *p* < .001), hopelessness (*r* = .22, *p* < .001), somatic symptoms (*r* = .27, *p* < .001), anxiety (*r* = .33, *p* < .001), loneliness (*r* = .33, *p* < .001), and anger (*r* = .34, *p* < .001), revealing that higher levels of distress concealment were related to greater mental illness symptoms.

**Table 3.** Zero-order correlations between personal characteristics and mental health indicators

	Age	Personal income	Employment status	Male-dominated workplace	Distress concealment
Depression	−.181***	−.026	−.029	.121*	.359***
Suicidal/self-injury ideation	−.195***	−.077	−.038	−.039	.228***
Hopelessness	−.078	−.139**	−.052	.010	.218***
Somatic symptoms	−.014	−.042	.022	.129**	.268***
Anxiety	−.199***	−.022	−.023	.059	.326***
Hazardous drinking	−.066	.162***	−.011	.051	.050
Loneliness	−.214***	−.088	−.020	.009	.327***
Anger	−.135**	−.052	−.055	.054	.338***

Note. *N* = 451; *p < .05, **p < .01, ***p < .001.

Burnout frequency was significantly associated with depression (*r* = .50, *p* < .001), anxiety (*r* = .49, *p* < .001), and suicidal/self-injury ideation (*r* = .32, *p* < .001), revealing higher rates of burnout frequency among those with greater mental illness symptoms. Burnout frequency was also associated with age (*r* = -.27, *p* < .001), employment status (*r* = -.23, *p* < .001), and the extent to which a workplace was male-dominated (*r* = -.14, *p* = .002), revealing higher rates of burnout among younger men, men in full-time employment, and those working in more male-dominated workplaces. As shown in Table 4, experiencing bullying, physical violence, threats of violence, and/or sexual harassment at work in the last 12 months was significantly associated with depression (*t* = 6.20, *df* = 126, *p* < .001), suicidal/self-injury ideation (*t* = -4.06, *df* = 116, *p* < .001), anxiety (*t* = 5.63, *df* = 132, *p* < .001), hopelessness (*t* = 2.09, *df* = 136, *p*=.04), somatic symptoms (*t* = 6.59, *df* = 123, *p* < .001), loneliness (*t* = 3.52, *df* = 449, *p* < .001), anger (*t* = 5.45, *df* = 449, *p* < .001), and burnout frequency (*t* = 5.75, *df* = 135, *p* < .001).

**Table 4.** Mental health indicators among men who experienced bullying, physical violence, threats of violence, and/or sexual harassment at work in the last 12 months

	Bullying, physical violence, threats of violence, and/or sexual harassment at work in the last 12 months					
	Yes (*n*=97)	No (*n*=354)				
	*M* (*SD*)	*M* (*SD*)	*F*	*t*	*p*-value	Cohen’s *d*
Depression	9.38 (7.04)	4.63 (5.18)	21.21	6.20	<.001	.84
Suicidal/self-injury ideation	0.60 (0.93)	0.19 (0.57)	62.29	4.06	<.001	.66
Anxiety	6.98 (5.48)	3.58 (4.41)	9.81	5.63	<.001	.73
Hazardous Drinking	3.25 (2.80)	2.78 (2.20)	8.27	1.51	.13	.20
Hopelessness	5.20 (2.44)	4.63 (2.06)	9.77	2.09	.04	.26
Somatic symptoms	10.81 (6.76)	5.99 (4.76)	29.80	6.59	<.001	.92
Loneliness	5.26 (1.99)	4.53 (1.75)	3.70	3.52	<.001	.40
Anger	12.84 (4.62)	10.27 (3.97)	3.86	5.45	<.001	.63
Burnout frequency	3.16 (1.95)	1.92 (1.62)	8.49	5.75	<.001	.73

## Discussion

While the prevalence and impacts of mental ill-health at work have received growing attention, research has predominantly been conducted on mixed-gender samples or men in male-dominated industries (Roche et al., 2016; Seaton et al., 2017). Our findings revealed high prevalence and diversity of men’s mental health challenges across a broad spectrum of workplaces/industries and point to some important contextualising factors (age, income, distress concealment, male-dominated workplace) that can help inform prevention and support strategies. These findings draw attention to the need for policy makers and employers to take immediate action by developing initiatives that support men’s mental health in the workplace. It is interesting that the mental health challenges reported in the current study were greater among younger working men. Young men were particularly susceptible to employment insecurity and experienced the greatest mental health burden during the pandemic (Gottert et al., 2022). Kimmel (2008) characterised young men’s life course milestones (i.e., marriage, family, career) as being significantly delayed when compared to their forefathers’ generations. It is entirely reasonable to suggest that COVID-19 further stalled (if not forever changed) young men’s progressions and trajectories, which may in part, explain the mental illness challenges identified here. Specific to work and career, young men have been especially challenged to comfortably work and live in British Columbia, a province where the average price of a house is a million dollars (Statista, 2023). With a growing divide between young men’s reality and the prospects of home ownership and financial freedom, the value of men’s labour has been drawn into question, especially in terms of what can be expected as the fruits of those toils. While historically Western masculine capital was based on purchasing power and material goods, the lack of leverage in many young men’s (under)paid work may well explain some of the mental health challenges reported here and elsewhere (Ruxton & Burrell, 2020; Walther et al., 2023). It may also be that young men are more likely to recognise their distress symptoms as mental health challenges and/or experience less mental health self-stigma, increasing the likelihood that they endorse the survey items.

Distress concealment was identified as an important characteristic, correlating with several mental health indicators (i.e., depression, suicidal/self-injury ideation, hopelessness, somatic symptoms, anxiety, loneliness, and anger). These findings align with previous research indicating men’s distress concealment was associated with feeling less understood by others, loneliness, and depressive symptoms (Cox et al., 2020). Similarly, Keum et al. (2023) reported that men who *disclosed* their distress to others felt more understood and less lonely, contributing to better psychological well-being. These findings regarding men’s distress concealment highlight the importance of workplace values and culture (e.g., psychological safety, trust) that support men’s mental health disclosures at work. Initiatives such as Buddy Up, a peer-based suicide prevention campaign for men, may be helpful to develop cultures that are conducive to distress disclosure and peer support for men’s mental health challenges (Sharp et al., 2023).

Many participants reported that they experienced occupational burnout, which was positively correlated with several mental health challenges. Participants’ understanding and perceptions of burnout may be influenced by normative North American workplace cultures that idealise high productivity, time pressure, and comparatively measure individual performance (Schaufeli, 2017). This relationship between men’s mental health and burnout is likely bidirectional and highlights the importance of policy makers and employers taking purposeful strides to address work-related factors contributing to burnout as well as offering mental health promotion to employees. Burnout is often assumed to be a negative consequence of chronic work stress and is an independent risk factor for depressive symptoms, work disability, decreased productivity, and presenteeism (Ahola, 2007; Salvagioni et al., 2017). Experiencing mental health challenges may increase men’s susceptibility to burnout (Yang & Hayes, 2020), as might diverse individual and work-related factors such as job demands, negative job attitudes, low adaptive coping, and limited leisure-time (Shoman et al., 2021). In the present study, higher rates of burnout were identified among younger men, men in full-time employment, and those working in more male-dominated workplaces. In addition, men who experienced workplace threats, violence, or harassment (over 20% of respondents) reported poorer mental health on many indicators (e.g., suicidality, depression, anxiety, burnout).

The workplace is an important arena for men’s mental health promotion and well-designed policy and practices can reach men who may not otherwise be accessing mental health care (Lee et al., 2014; Robertson et al., 2015). As many organizations have existing infrastructure and frameworks to support employees’ health, mental health promotion sits neatly within the scope of occupational health and safety programs. While employers might offer a suite of resources including mental health education, employee assistance programs, or counselling services, efforts must acknowledge and address masculine workplace cultures that limit men’s mental health promotion (Seaton et al., 2019). Failing to shift perceptions and expectations of men’s commitment to work are likely to contradict and erode any accrued benefits (Lashewicz et al., 2020). These considerations may be particularly salient in the context of countries like the UK, USA, and Australia trialing a four-day work week as a means to address workplace stress and improve work–life balance (4dayweek.com, 2023). While these trends are important steps, there is a need to thoughtfully evaluate and respond to the many cause-effects of such changes. Although the degree to which a workplace was male-dominated was associated with only a limited number of mental health indicators, this does not negate the need to target these locations as sites where traditional masculinities are often upheld and defended more vigorously. Evidence suggests that workplace interventions tailored to men’s needs and interests have the potential to produce significant improvements in men’s psychosocial health and well-being (Seaton et al., 2017).

Limitations of this study must be considered. Generalizability is likely to be limited and replication of these findings across other provinces and countries is needed. However, participants were sourced from various regions (e.g., urban and remote) and sectors, without knowing the context of the study. The cross-sectional and exploratory nature of the study does not allow for directionality to be examined; thus the reported relationships must be regarded as preliminary. Additionally, the use of self-report screening tools and cut-off thresholds may contribute to an overestimation of mental health challenges. This limitation can be addressed through the use of diagnostics interviews in future research (Thombs et al., 2018). Analyses were performed ad hoc and should be considered with appropriate caution. Future studies might examine the influence of relevant covariates (e.g., workload, shift work) to identify priority populations for targeted mental health promotion efforts. Additionally, longitudinal research is needed to further examine potential shifts in the relationship between men’s work and various mental health indicators.

In conclusion, it is clear that men’s workplace mental health is both an area of concern and opportunity. Findings revealed the high prevalence and diverse nature of mental health challenges experienced by Canadian men who participated in this study. The findings help to identify vulnerable sub-groups of men in need of targeted interventions, including younger men and men with high levels of distress concealment. Future efforts must be cognisant of working with men’s masculine and work-related identities to re-imagine new and flexible gendered ways for men to effectively navigate ever-present global and economic uncertainties.
